# Giant Osteofibrous Dysplasia (Ossifying Fibroma) of the Tibia: Case Report and Review of Treatment Modalities

**DOI:** 10.1080/13577140410001679275

**Published:** 2004-03

**Authors:** Eitan Segev, Josephine Issakov, Eli Ezra, Shlomo Wientroub, Itzchak Meller

**Affiliations:** 1 Department of Pediatric Orthopaedics Dana Children Hospital Tel-Aviv Medical Center 6 Weizmann Street Tel Aviv 64239 Israel; 2 The National Unit of Orthopaedic Oncology Tel-Aviv Medical Center Sackler Faculty of Medicine Tel Aviv University Tel Aviv Israel; 3 The Department of Pathology Tel-Aviv Medical Center Sackler Faculty of Medicine Tel Aviv University Tel Aviv Israel

## Abstract

We present a case of giant osteofibrous dysplasia (OFB) of the proximal tibia with 15 years of follow-up. The tumor recurred after first biopsy and curettage done at the age of 6 years and, again, 4 years later. Following recurrence, the option of amputation was suggested. Staged treatment of curettage, cryosurgery, bone cement as a temporary spacer with internal fixation at age 12 years, followed by bone grafting at age 14 years, led to complete healing. The staged protocol for treatment is proposed as an alternative to more radical solutions. It is suggested to postpone surgical treatment towards skeletal maturity.


*Sarcoma*, March 2004, *VOL. 8, NO. 1, 51–56*

Taylor & Francis healthsciences

CASE REPORT

### Giant osteofibrous dysplasia (ossifying fibroma) of the tibia: case report and review of treatment modalities

EITAN SEGEV[1], JOSEPHINE ISSAKOV[3], ELI EZRA[1], SHLOMO WIENTROUB[1] & ITZCHAK MELLER[2]

[1]*Department of Pediatric Orthopaedics, Dana Children's Hospital,* [2]*The National Unit of Orthopaedic Oncology,* [3]*The Department of Pathology, Tel-Aviv Medical Center & Sackler Faculty of Medicine, Tel Aviv University, Tel-Aviv, Israel*

**Abstract**

We present a case of giant osteofibrous dysplasia (OFB) of the proximal tibia with 15 years of follow-up. The tumor recurred after first biopsy and curettage done at the age of 6 years and, again, 4 years later. Following recurrence, the option of amputation was suggested. Staged treatment of curettage, cryosurgery, bone cement as a temporary spacer with internal fixation at age 12 years, followed by bone grafting at age 14 years, led to complete healing. The staged protocol for treatment is proposed as an alternative to more radical solutions. It is suggested to postpone surgical treatment towards skeletal maturity.

**Key words:** *osteofibrous dysplasia, ossifying fibroma, cryosurgery*

### Introduction

Campanacci described osteofibrous dysplasia (OFB) of long bones as a distinct entity in 1976.[1] Additional synonyms are: ossifying fibroma, congenital osteitis fibrosa cystica, congenital fibrous dysplasia, congenital fibrous defect of the tibia and intracortical fibrous dysplasia of the tibia.[2–4]

The tumor may appear as early as birth and up to the end of the second decade of life. The lesion unifocal or multifocal involves and expands the cortices of the shaft of the tibia and occasionally of the fibula and may cause a bowing deformity. Reports on OFB of other bones such as radius and ulna are rare.[5]

The typical radiographic appearance is of multiple expansile cortical lytic lesions with a zone of sclerosis around them. The radiological differential diagnosis includes non-ossifying fibroma, monostotic fibrous dysplasia and adamantinoma.[6]

The lesion is composed of loose fibrous tissue with focal storiform pattern and woven bone trabeculae rimmed by osteoblasts. Zonal architecture with progressive maturation and increased mineralized bone trabeculae in the periphery are seen. Similarity to adamantinoma of the tibia has been described in some cases with histological evidence of keratin-positive epithelial elements.[7]

OFB has an unpredictable behavior: 'congenital' and infantile cases tend to regress spontaneously during the first years of life, while later appearing lesions have a tendency to progress and recur after curettage and bone grafting.[8,9] The recommendation in the literature is to avoid surgery in the first decade of life and to perform resection if necessary in the second decade.[9]

We present a case of a symptomatic extensive giant OFB of the tibia. The large tumor recurred after two surgical procedures in the first decade of life. Curettage and cryosurgery done at the age of 12 years, followed by staged reconstruction of the bone without damaging the epiphyseal plate, resulted in healing of the lesion. Cryosurgery and the staged reconstruction may be an alternative approach to more radical ways such as amputation or endoprosthetic reconstruction.

### Case report

F.O. (male patient) was born in 01/1980, the result of uneventful pregnancy and normal delivery. The family history revealed one sister being followed-up for a benign cystic lesion of a rib. He had no medical problems until the age of 6 years, when he presented

Correspondence to: E. Segev, M.D., Department of Pediatric Orthopaedics, Dana Children Hospital, Tel-Aviv Medical Center, 6 Weizmann Street, 64239 Tel Aviv, Israel. Tel.: +972-3-6974261; Fax: +972-3-6974542; E-mail: esegev@tasmc.health.gov.il

1357-714X print/1369–1643 © 2004 Taylor & Francis Ltd
DOI: 10.1080/13577140410001679275

to our department with a 2-month history of rapidly growing solid tumor in the left tibia extending from the metaphysis into the proximal diaphysis. The size of the tumor at presentation was 4 × 4 cm. The radiographic appearance was of an intra-cortical cystic lesion with a sclerotic reactive rim. Anterior bowing of the tibia was evident (Fig. 1). The patient underwent the following staging studies: three-phase Tc-99 total body bone scan showed increased uptake only in the left proximal tibia; CT scan of the chest was normal; ultrasound of the abdomen was normal. In May 1986, through a longitudinal antero-lateral approach fenestration, biopsy and curettage were performed. The cavity was filed with autologous bone graft (Fig. 2). A pathological review opinion raised the possibility of low grade osteosarcoma due to the presence of atypical mitotic activity in some of the microscopic fields; hence the treatment advice was amputation. The final pathological conclusion was of osteofibrous dysplasia (OFB). The clinical decision was to continue with close follow-up of the patient.

During the next 4 years the tumor continued to expand slowly and the possibility of secondary aneurysmal bone cyst formation was raised. At the age of 10 years, an open excisional biopsy was performed. A lump of 7 cm in circumference was removed from the bed of the tumor. The pathology report was osteofibrous dysplasia (OFB) of bone.

At the age of 11 years, the patient underwent angiography of the leg which showed increased vascularity of the tumor area. The possibility of treating the tumor with embolization was considered but rejected because of high risk of distal leg necrosis.

The tumor kept growing and reached 12 × 15 × 17 cm in size (Figs. 3 and 4). At the age of 12, an intralesional resection was done. The cavity walls were curetted and treated with cryosurgery (open system technique using liquid nitrogen according to Marcove[10]). The space was filled with the bone cement polymethylmethacrylate (PMMA), and the bone was stabilized by an intramedullary rod reinforced with side plates, screws and wires as a temporary spacer (Fig. 5). Pathology report was of recurrent ossifying fibroma of bone with secondary aneurysmal bone cyst. Review of the histological slides reveals fragments of bone and fibrous tissue. The fibrous tissue is loose, composed of spindle cells with storiform pattern and myxoid areas and immature bone trabeculae (Fig. 6). The trabeculae are lined by osteoblasts (Fig. 7). In the periphery, the trabeculae anastomose and merge with the thickened cortex in a zonation phenomenon. Part of the material represented a secondary aneurismal bone cyst (Fig. 8). An observation period of 30 months did not reveal evidence of local recurrence. At the age of 14 years, the cement and hardware were removed and the space filled with autologus bone graft reinforced by an intra-medullary nail.

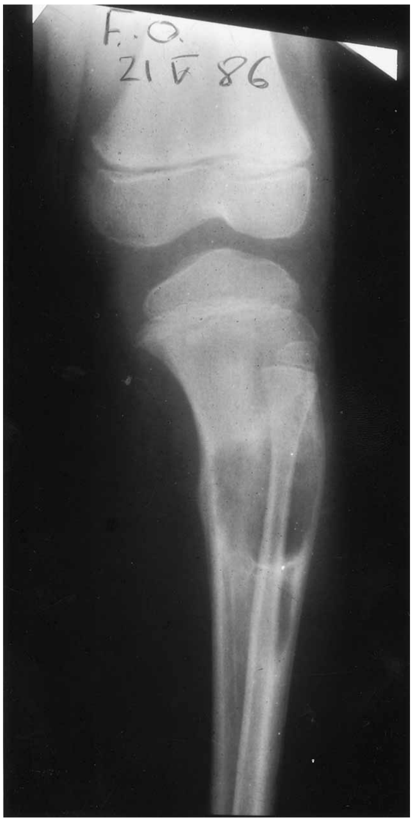

**Fig. 1.** *Radiograph of proximal left tibia at presentation, there is intra-cortical cystic lesion with sclerotic reactive rim. There is anterior bowing of the bone.*

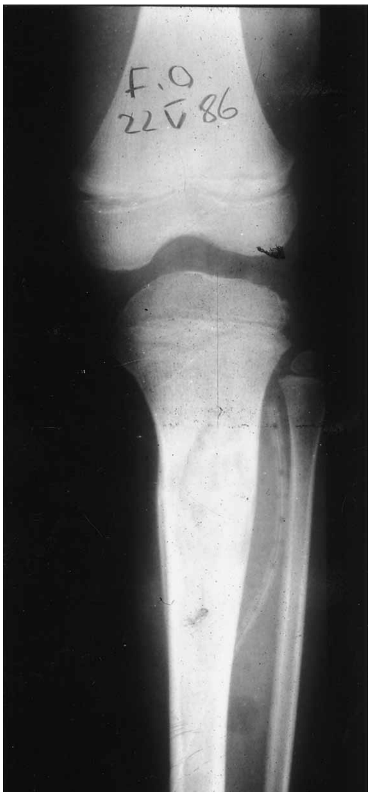

**Fig. 2.** *Postoperative radiograph, the cavity is filled with autologous bone marrow.*

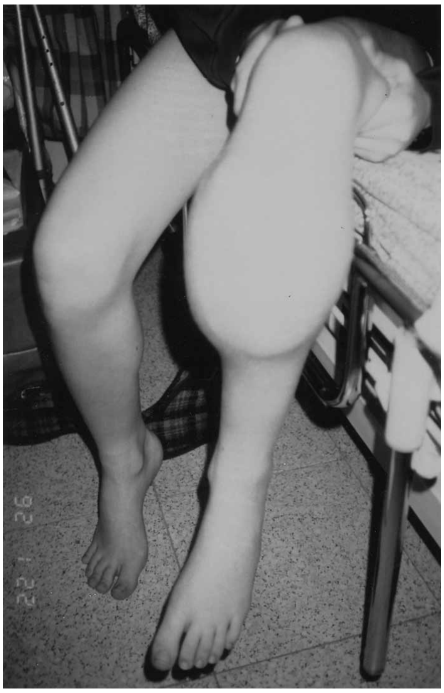

**Fig. 3.** *Clinical appearance of the tumor before resection at age 12 years, the tumor size is of a baby's head.*

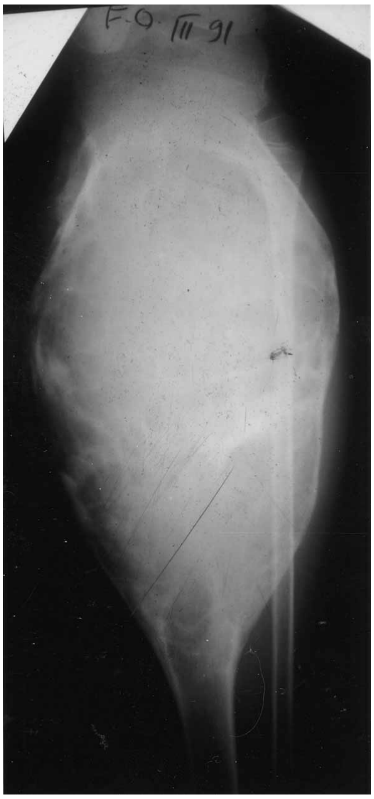

**Fig. 4.** *Radiographic appearance of the tumor before resection, there is a distinctive cystic nature with lytic areas and sclerotic rim.*

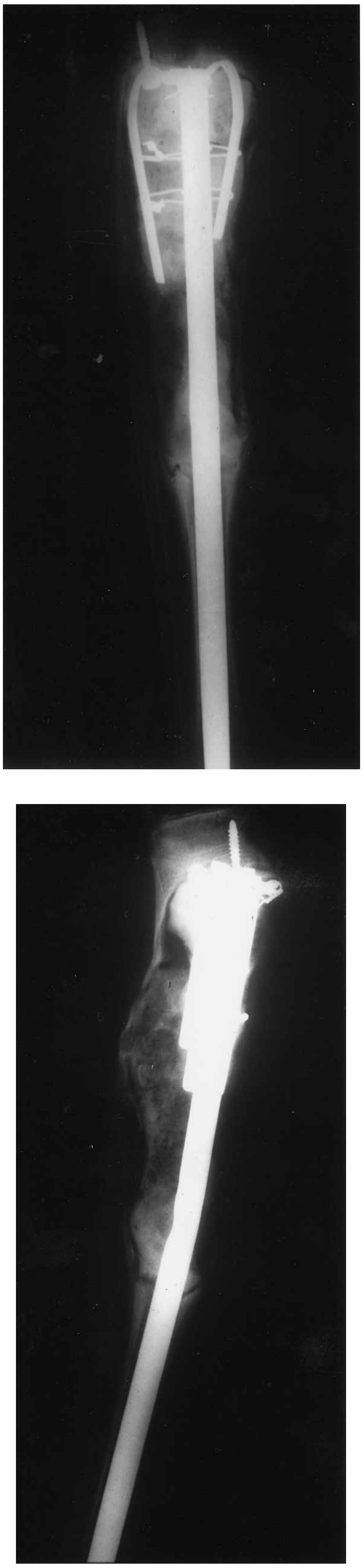

**Fig. 5.** *Anterior and lateral radiograph after curettage and cryosurgery; the cavity is filled with bone cement and reinforced by intramedullary nail and plates.*

Follow up of 7 years showed excellent incorporation of the bone graft with subperiosteal bone formation and no evidence of local recurrence, the proximal tibial growth plate was unaffected. There was a complete reconstruction of the affected tibia. On last follow-up, the patient was 21 years old, has no pain and enjoys unrestricted daily sport activities. Physical examination 15 years from first presentation

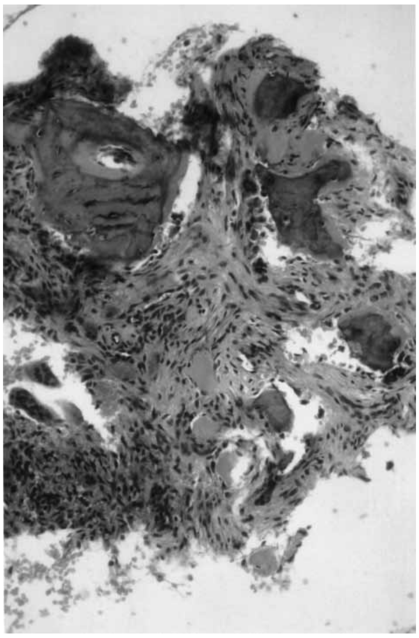

**Fig. 6.** *Histology specimen: there is loose fibrous tissue, composed of spindle cells with storiform pattern and myxoid areas. Immature bone trabeculae can be observed.*

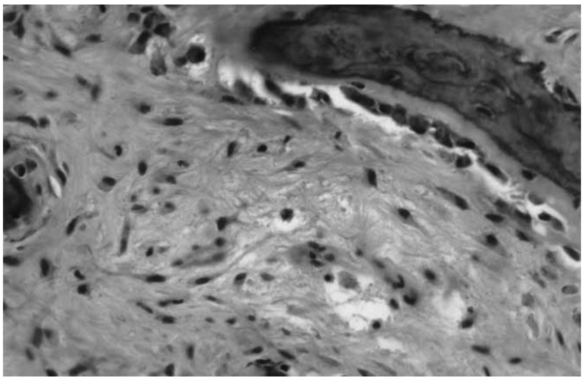

**Fig. 7.** *Histology specimen: the bony trabeculae are lined by osteoblasts.*

reveals equal leg length with full knee and ankle range of motion (Figs. 9 and 10).

### Discussion

Reviewing the literature concerning treatment of OFB it is evident that age at presentation is the major decisive factor. In cases described as congenital or infantile, with typical histological appearance, the lesion tends to regress and will not cause clinical symptoms.[8] Surgery in this group is recommended for increasing bowing deformity or for very large masses.[2,8,11] Additional indication for surgery is an expanding lesion that involves the whole of the bone; such a rare lesion involving the ulna has been described.[5]

Another concern when dealing with this tumor is its association with adamantinoma. Adamantinoma is a rare malignant lesion and may cause distant metastasis, it appears mainly in the second decade of life, and the treatment is wide resection. The expanding lytic aggressive intracortical lesions resemble adamantinoma radiographically. Case reports that described histological specimens from OFB with areas of islands of epithelioid cells further

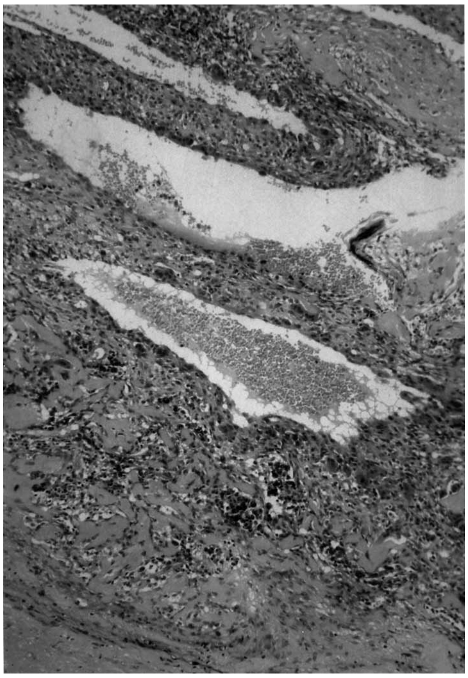

**Fig. 8.** *Histology specimen: material representing features of a secondary aneurysmal bone cyst.*

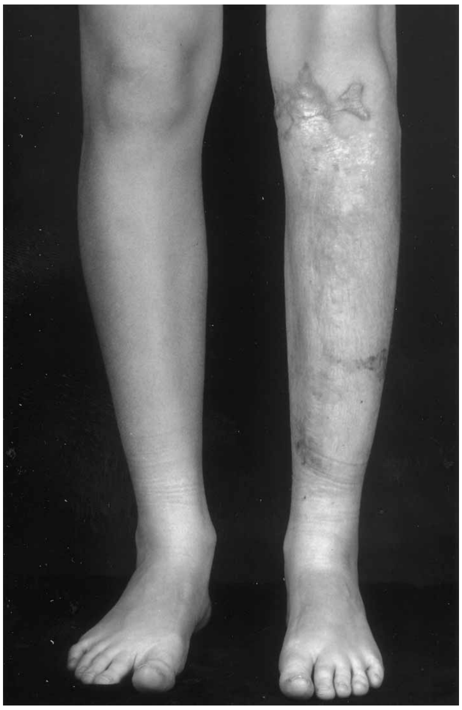

**Fig. 9.** *Anterior and lateral clinical pictures of the left tibia, the patient is 21 years old and 7 years after the last surgery, no tumor recurrence.*

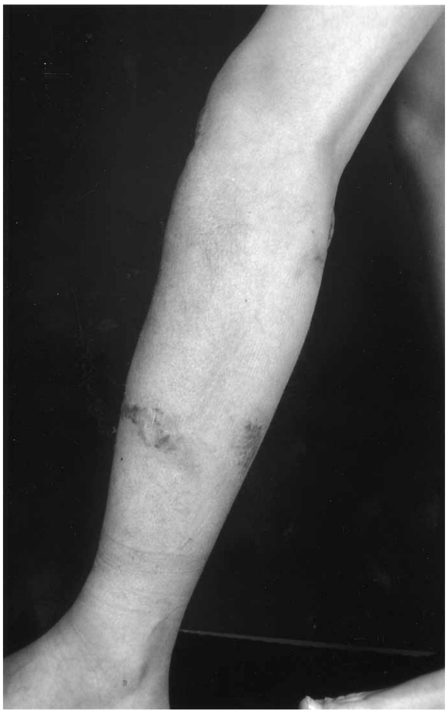

**Fig. 9.** *Continued.*

support this similarity.[6,12] There are reports of tibial adamantinoma with features similar to OFB, which may represent a 'differentiated' type of lesion.[7] A retrospective study of biopsy specimen from patients diagnosed as OFB and needed resection revealed features of adamantinoma in six of 10 patients; however, there is no documentation of OFB transforming into adamantinoma.[3,13]

The good prognosis of OFB merits observation before 10 years of age; however, the association with adamantinoma may require biopsy at presentation mainly in the second decade of life.[4,14] Literature review of OFB establishes its unique intra-cortical location, its histological pattern that is easily distinguishable from monostotic fibrous dysplasia and the tendency for recurrence after curettage.[15]

The paper by Campanaccci *et al.*[9] that described the natural history of OFB as a self-limiting disease with a tendency to spontaneous regression, does not support limited surgical treatment such as fenestration and curettage or resection due to high recurrence rate.[16.] This tendency for recurrence in young age and good results obtained in surgery done after age 15 years led to the recommendation to perform the curettage near skeletal maturity.[4]

Our patient had giant OFB with a significant deformity of the tibia. His first curettage was done at age 6 years. As may be expected the tumor recurred and progressed rapidly and another debulking biopsy was done at age 10 years. The tumor kept growing to giant proportions. Fenestration and excision of the anterior cortical shell, intralesional curettage, burr drilling and cryosurgery were done. Reconstruction of the defect included polymethylmethacrylate bone cement (PMMA) and intramedullary device with preservation of the epiphyseal plate. This was supposed to serve as a temporary spacer for about 2 years in order to see that there is no local recurrence.

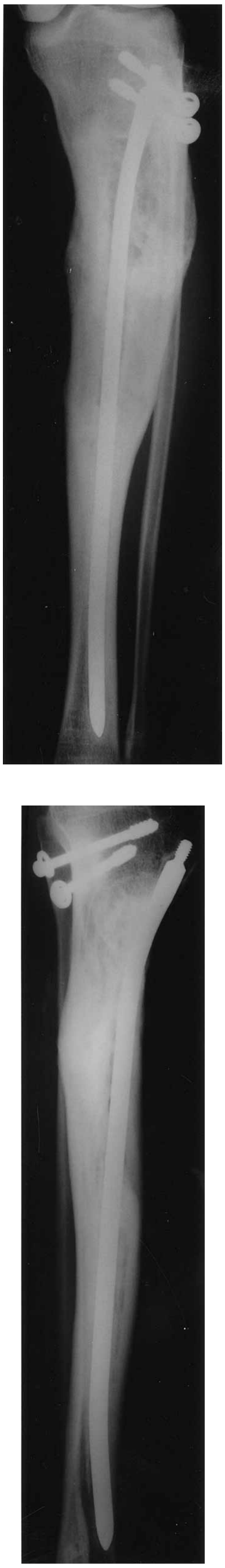

**Fig. 10.** *Anterior and lateral radiograph of last follow-up, there is incorporation of the bone graft around the intramedullary nail and no local recurrence.*

Removal of the spacer and filling of the defect with autologous bone graft and intramedullary nailing was performed 2 years later with no more recurrences.

We believe that, if the indication arises, giant OFB in the first decade of life can be treated successfully by a staged procedure. In the first stage the tumor is curetted and cryosurgery is used for total eradication and prevention of local recurrences. Cryosurgery is very effective in our experience for the prevention of local recurrence in other more common tumors.[17,18] For mechanical support, an internal fixation with bone cement (PMMA) as a spacer can be used; when follow-up establishes no tendency for recurrence, final surgery with bone graft and internal fixation can be performed. For our patient and in similar cases this approach may spare the patient radical solutions such as amputation.

### References

1. Campanacci M. Osteofibrous dysplasia of long bones: A new clinical entity. *Ital J Orthop Traum* 1976; 2: 221–37.
2. Anderson MJ, Townsend DR, Johnston JO, Bohay DR. Osteofibrous dysplasia in the Newborn. *J Bone Jt Surg* 1993; 75-A(2): 265–7.
3. Sweet DE, Vinh TN, Devaney K. Cortical osteofibrous dysplasia of long bone and its relationship to adamantinoma. A clinopathologic study of 30 cases. *Am J Surg Pathol* 1992; 16(3): 282–90.
4. Wang J-W, Shih C-H, Chen W-J. Osteofibrous dysplasia (ossifying fibroma of long bones). *Clin Orthop Rel Res* 1992; 278: 236–43.
5. Kamineni S, Briggs TWR, Saifuddin A, Sandison A. Osteofibrous dysplasia of the ulna. *J Bone Jt Surg* 2001; 83-B(8): 1178–80.
6. Markel SF. Ossifying fibroma of long bone: its distinction from fibrous dysplasia and its association with adamantinoma of long bone. *Am J Clin Pract* 1978; 91–7.
7. Czerniak B, Rojas-Corona RR, Dorfman HD. Morphologic diversity of long bone adamantinoma. The concept of differentiated (regressing) adamantinoma and its relationship to osteofibrous dysplasia. *Cancer* 1989; 64: 2319–24.
8. Komiya S, Inoue A. Aggressive bone tumorous lesion in infancy: osteofibrous dysplasia of the tibia and fibula. *J Pediatr Orthop* 1993; 13: 577–81.
9. Campanacci M, Laus M. Osteofibrous dysplasia of the tibia and fibula. *J Bone Jt Surg* 1981; 63-A(3): 367–75.
10. Marcove RC. A 17-year review of cryosurgery in the treatment of bone tumors. *Clin Orthop Rel Res* 1982; 163: 231–4.
11. Semian DW, Willis JB, Bove KE. Congenital fibrous defect of the tibia mimicking fibrous dysplasia. *J Bone Joint Surg* 1975; 57-A(6): 854–7.
12. Alguacil-Garcia A, Alonso A, Pettigrew NM. Osteofibrous Dysplasia (ossifying fibroma) of the tibia and Fibula and adamantioma. *Am J Clin Pract* 1984; 82(4): 470–4.
13. Springfield DS, Rosenberg AE, Mankin HJ, Mindell ER. Relationship between osteofibrous Dysplasia and adamantinoma. *Clin orthop Rel Res* 1994; 309: 234–44.
14. Van Delm I, Fabry G. Osteofibrous Dysplasia of the tibia: case report and review of the literature. *J Pediatr Orthop* 1999; 8-B: 50–3.
15. Goergen TG, Dickman PS, Resnick D, Saltzstein SL, O'Del CW, Akeson WH. Long bone ossifying fibromas. *Cancer* 1977; 39: 2067–72.
16. Campbell CJ, Hawk T. A variant of fibrous Dysplasia (osteofibrous dysplasia). *J Bone Jt Surg* 1982; 64-A(2): 231–5.
17. Bickels J, Meller I, Shmookler BM, Malawer MM. The role and biology of cryosurgery in the treatment of bone tumors – a review. *Acta Orthop Scand* 1999; 70: 308–15.
18. Segev E, Kollender Y, Bickels J, Flusser G, Issakov J, Wientroub S, Meller I. Cryosurgery in fibrous dysplasia: good result of a multimodality protocol in 16 patients. Technical note. *Acta Orthop Scand* 2002; 73(4): 483–6.
